# Nuclear Mechanics in the Fission Yeast

**DOI:** 10.3390/cells8101285

**Published:** 2019-10-20

**Authors:** Paola Gallardo, Ramón R. Barrales, Rafael R. Daga, Silvia Salas-Pino

**Affiliations:** Centro Andaluz de Biología del Desarrollo, Universidad Pablo de Olavide-Consejo Superior de Investigaciones Científicas, Junta de Andalucia, 41010 Seville, Spain; pgalpal@alu.upo.es (P.G.); rrambar@upo.es (R.R.B.)

**Keywords:** nucleus, genome 3D organization, nuclear architecture, nuclear envelope, inner nuclear membrane (INM) proteins, linker of nucleoskeleton and cytoskeleton (LINC) complex, chromatin, chromatin domains, microtubule (MT) cytoskeleton, MT pushing forces, nuclear mechanics

## Abstract

In eukaryotic cells, the organization of the genome within the nucleus requires the nuclear envelope (NE) and its associated proteins. The nucleus is subjected to mechanical forces produced by the cytoskeleton. The physical properties of the NE and the linkage of chromatin in compacted conformation at sites of cytoskeleton contacts seem to be key for withstanding nuclear mechanical stress. Mechanical perturbations of the nucleus normally occur during nuclear positioning and migration. In addition, cell contraction or expansion occurring for instance during cell migration or upon changes in osmotic conditions also result innuclear mechanical stress. Recent studies in *Schizosaccharomyces pombe* (fission yeast) have revealed unexpected functions of cytoplasmic microtubules in nuclear architecture and chromosome behavior, and have pointed to NE-chromatin tethers as protective elements during nuclear mechanics. Here, we review and discuss how fission yeast cells can be used to understand principles underlying the dynamic interplay between genome organization and function and the effect of forces applied to the nucleus by the microtubule cytoskeleton.

## 1. Introduction

Eukaryotic cells show a complex nuclear organization that has co-evolved along with increasing genome size and complexity. Nuclear organization is essential to many aspects of genome regulation and stability. Regulation of gene expression, DNA replication and repair, and ribosome synthesis depend on positioning and complex interaction of DNA and proteins within the nucleus [1,2,3,4]. The recent rapid development of the nuclear architecture field has established the basis for understanding overall genome organization from yeast to human, and has provided mechanisms and structures contributing to this organization [2,5,6,7,8,9,10,11].

An important element in the 3D organization of the nucleus is the nuclear envelope (NE). The NE, inner nuclear membrane (INM) proteins, and other NE-associated complexes such as linker of nucleoskeleton and cytoskeleton (LINC) complexes, nuclear pore complexes (NPCs), or nuclear lamina in animal cells provide a binding platform for specific DNA sequences and chromatin, creating specialized chromatin domains and contributing to the 3D organization of the genome [12,13,14,15,16]. In addition to its function as overall spatial genome organizer, the NE and its tethered chromatin domains are important as they provide structural support to the nucleus during mechanical stress [17]. In the last few years, the mechanics of the nucleus have attracted much attention as it has been demonstrated that the nucleus is a mechanosensitive organelle that is able to sense mechanical inputs through the NE and its associated structures, and to transduce these inputs into a biological response at the level of chromatin regulation and gene expression [18,19,20,21]. 

The fission yeast *Schizosaccharomyces pombe* has been established as an excellent model organism for the study of conserved principles underlying eukaryotic nuclear organization (reviewed in [6,7,8,9]). *S. pombe* is a rod-shaped cell that has a small genome (13.8 Mb) with just three chromosomes and a relatively large nucleus, which is suitable for microscopic observation. Its chromatin organization and regulation share many features with that of higher eukaryotes [6,7,8,9,22,23,24], and several INM proteins contribute to support this nuclear organization [25,26,27]. 

**Figure 1:**
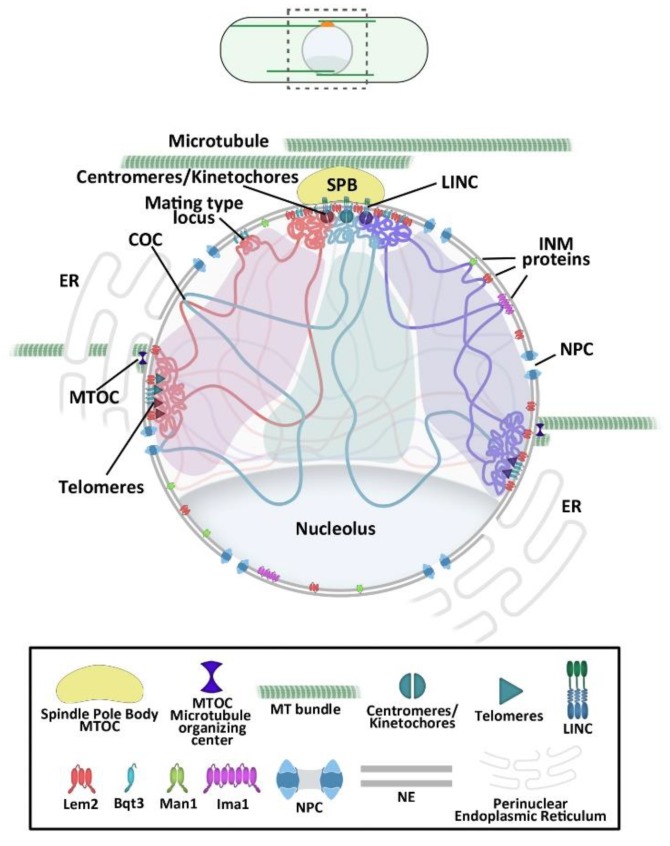
Schematic representation of the fission yeast nucleus. Schematic representation of a fission yeast cell (above). Magnification of the area marked by dashed lines (below). Global chromosome organization with centromeres attached underneath the spindle pole body (SPB) and telomeres and nucleolus distantly positioned. Chromatin is linked to the nuclear envelope (NE) by the interaction of different genomic elements with inner nuclear membrane (INM) proteins and linker of nucleoskeleton and cytoskeleton (LINC) complexes. Note that the interaction of Lem2 protein with chromatin might be indirect. The NE is continuous with the perinuclear endoplasmic reticulum. The SBP and other microtubule organizing centers (MTOCs) organize antiparallel bundles of microtubules (MTs).

The fission yeast cytoplasmic microtubule MT cytoskeleton is relatively complex [28] and, among other functions, is responsible for nuclear positioning at the cell center [29]. During interphase, the spindle pole body (SPB, centrosome equivalent), the main MT organizing center (MTOC), is attached to the cytoplasmic surface of the NE, from where it nucleates antiparallel cytoplasmic MT bundles. In addition to the SPB bundles, several other MTs are nucleated from MTOCs and recruited to NPCs along the NE surface [30] (Figure 1). These cytoplasmic MTs, with their plus ends facing the cell tips, alternate between cycles of growth and shrinkage. When a MT bundle contacts a cell tip, it keeps polymerizing, producing pushing forces that are transmitted along the MT bundle to the NE and results in the movement of the nucleus. Alternated cycles of MT polymerization at each cell tip dynamically positions the nucleus at the cell center [29,31] and this is in turn essential for proper cell division plane positioning [31]. As cytoplasmic MTs are connected to chromatin through LINC complexes at the SPB and to other sites at the NE [28,30], these MT-generated forces not only contribute to nuclear positioning, but are also transmitted to the chromatin inside the nucleus [29,31,32,33,34,35,36].

In this review, we will first provide a brief overview of global nuclear organization in the fission yeast with a focus on the role of the NE and its associated elements as genome organizers. Then, we will highlight the latest findings on the role of cytoplasmic MTs on chromosome dynamics, the modulation of the DNA damage response, homologous recombination (HR), and chromatid cohesion. Finally, we will discuss how this overall nuclear organization is relevant to bear mechanical forces produced by cytoplasmic MTs.

## 2. Overall Chromosomal Organization in the Fission Yeast

**Figure 2:**
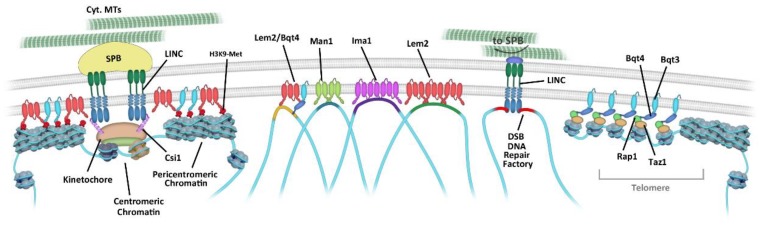
NE microdomains and their association with different genomic regions. Schematic representation of the linkage between centromeres, telomeres, double strand breaks (DSBs) and other genomic loci and INM proteins. DSB repair factories are linked to the NE by the LINC complex and can be moved by cytoplasmic MTs and positioned in close proximity of the SPB. The different elements are depicted as in Figure 1.

In *S. pombe*, the three chromosomes are organized in a Rabl-like configuration in which centromeres are clustered and attached to the NE beneath the SPB, and telomeres are attached to distant sites in the NE opposite to the SPB [37] (Figure 1). The mating-type locus is positioned at the nuclear periphery close to centromeres [38,39]. The ends of chromosome III that harbor the rDNA repeats organize the nucleolus, a differentiated nuclear territory where rRNA genes are expressed and ribosomes are preassembled [40] (Figure 1). This configuration of chromosomes observed during interphase is the result of multiple interactions between several INM proteins and LINC complexes with specific chromatin domains [6] (Figure 2). In addition, chromatin-NE interactions at multiple other loci, including polymerase III (pol III)-transcribed genes such as tRNAs and 5sRNA genes, or Long Terminal Repeats (LTR) of retroviruses within the fission yeast genome have been identified [27,41,42,43,44,45]. DNA adenine methyltransferase identification (DamID) studies have shown that INM proteins such as Ima1 and the Lap-emerin-Man1 (LEM)-domain containing protein Man1, interact with multiple loci that are mostly heterochromatic across the *S. pombe* genome. Man1 has a broad interaction map that spans about a third of the genome and its interacting domains are enriched for Swi6/HP1, a hallmark of heterochromatin [27]. Ima1 has a less extensive interaction profile compared to Man1 and it is more specific for loci enriched for the components of the iRNA silencing pathway, namely, Dcr1 and Rdp1 [27]. Thus, the NE through its INM proteins acts as a scaffold for chromatin, creating constraints for its free displacement and contributing to the spatial conformation of the fission yeast chromosomes within the nucleus.

## 3. The NE as Genome 3D Organizer

In the last years, multiple studies have demonstrated that the nuclear periphery constitutes a silenced environment where heterochromatin is promoted (Reviewed in [7,46]). Centromeres and telomeres assemble big blocks of heterochromatin that tether to the NE. In *S. pombe*, genome-wide chromatin contact maps have shown that heterochromatin at centromere-proximal regions promotes inter- and intra-chromosomal interactions, while it avoids contacts between centromere-proximal regions and chromosome arms [47]. This results in collinear extension of chromosomes at these regions. At euchromatic chromosome arms, basic chromatin organization is driven by repetitive, locally self-interacting domains of about 100 kilobases named “chromatin globules”, which are isolated from each other and from other regions of the genome by cohesins [44,47] (reviewed in [8,9]). Thus, the presence of heterochromatin along with centromere tethering immobilizes these regions of the chromatin and avoids ectopic chromatin contacts.

### 3.1. Centromere Attachment to the NE

In *S. pombe*, centromere attachment to the NE depends on the centromere-bound protein Csi1 and the LINC complex [48] (Figure 2). The LINC complex, is formed by the INM Sad1/UNC-84 (SUN) protein Sad1, which associates with the outer nuclear membrane (ONM) Klarsicht/ANC-1/Syne homology (KASH) domain-containing proteins, Kms1 and Kms2. At the cytoplasmic side, Kms2 interacts with the SPB, whereas at the nucleoplasmic side, Sad1 interacts with the centromere through Csi1 [48,49,50,51,52]. Csi1 bridges centromeres to the SBP/LINC complex through interaction of its N-terminal domain with Sad1 and the interaction of an internal coiled-coil domain with the kinetochore [48]. The lack of centromere attachment to the NE/SPB leads to severe defects in chromosome segregation [48,49]. In addition to Csi1 and LINC complexes, INM proteins such as the LEM-domain containing protein Lem2 have also been shown to contribute to centromere attachment to the NE (Figure 2) [53]. *S. pombe* Lem2 localizes at the NE and is also concentrated underneath the SPB in a Csi1-dependent manner, where it has a role in centromere positioning at the nuclear periphery and also in centromeric chromatin silencing [53,54,55]. Accordingly, the double mutant *lem2Δcsi1Δ* shows severe centromere detachment and defective pericentromeric gene silencing [53]. The tethering function of Lem2 is mediated by its N-terminal LEM domain, whereas the silencing function is mediated by its C-terminal MAN1/Src1 (MSC) domain. Thus, the same INM protein Lem2 displays separate roles, tethering and silencing, with regard to the centromeres [53,56,57].

### 3.2. Telomere Tethering to the NE

Telomere tethering to the NE also depends on several INM proteins such as Lem2, Man1, Ima1, and Bqt4 (Figure 2) [24,53,55,58,59,60]. Bqt4 recruits telomeres to the NE by binding to the telomere protein Rap1 [59]. This association occurs preferentially during the replication of telomeric sequences [42]. In addition, Bqt4 is essential for the correct localization of Lem2 along the NE surface. In the absence of Bqt4, Lem2 accumulates just beneath the SPB [42,61]. Telomere attachment does not greatly affect subtelomeric silencing, but NE-tethering is instead required for proper replication of these heterochromatic regions at the NE [42,59,62].

### 3.3. INM Microdomains

In *S. pombe* the distribution of INM proteins is not homogeneous along the NE surface but instead the NE presents specialized functional microdomains exclusively enriched in Man1, Lem2 or Bqt4. Lem2 enriched microdomains are concentrated at the SPB and are required for kinetochore maintenance [42,48] (Figure 2). Bqt4 microdomains are required for replication of telomeres and mating-type locus and Bqt4-Lem2 microdomains are involved in pericentromeric silencing and maintenance [42]. The function of Man1 microdomains has remained more elusive; however, evidences point to a role of Man1 in linking transcription boundaries to the nuclear periphery [58].

### 3.4. Transcription Boundaries

Transcription boundaries or boundary elements (BEs) are genomic positions that function by isolating heterochromatin domains from the surrounding euchromatin (reviewed in [63]). BEs are characterized by the presence of multiple B-boxes that are binding sequences for the transcription factor for polymerase IIIC (TFIIIC). TFIIIC and RNA pol III bind B-boxes and initiate the assembly of transcription complexes [44,58,64,65]. In *S. pombe* BEs are recruited at the NE [66,67], and they include LTRs at subtelomeric regions and RNA pol III-transcribed genes such as 5sRNA and tRNA genes at centromeres. Additional RNA pol III genes and LTRs dispersed throughout the genome cluster at the nuclear periphery, close to centromeres by the action of condensins that function as molecular connectors among chromatin fibers [33,43,66,67,68]. Man1 recruits tRNAs and LTRs to the NE [45,58] through interaction with the SNF2 chromatin-remodeling factor Fft3 [58]. The *S. pombe* genome presents other sites in which TFIIIC recruitment is independent of RNA pol III, such as the inverted repeats (IRs) of the mating-type locus and other dispersed TFIIIC sites [44]. These extra TFIIIC sites (ETCs) are thought to function as chromosome organizing clamps (COCs), that tether and cluster distant loci at the NE and partition the genome [44]. To date, how COCs/ETCs are tethered to the nuclear periphery in *S. pombe* is still unknown; however, in *S. cerevisiae*, ETCs peripheral localization depends on the LINC complex component Mps3 [69]. 

The functional significance of the interaction between the INM proteins and chromatin is an on-going field of study where many advances have been achieved using *S. pombe* as model system. INM proteins tether chromatin at multiple sites immobilizing these regions and also have a role in the regulation of heterochromatin. In the last few years, it has been demonstrated that the state of chromatin impacts the mechanical response of the nucleus. Condensed chromatin provides rigidity to the nucleus and metazoan cells can regulate the level of chromatin compaction in order to increase the resistance of the nucleus in conditions of mechanical stress [70,71,72,73,74,75].

## 4. Nuclear Organization is Necessary to Support Nuclear Mechanics

Cells are constantly subjected to mechanical stress in nature, which has profound repercussions not only in cell shape and morphology, but also in nuclear architecture. Nuclear architecture is in turn essential to resist nuclear mechanical stress. Tension, shear stress, or changes in pressure generate mechanical forces that are transmitted to the nucleus and this affects nuclear positioning, shape, and function. In *S. pombe*, cytoplasmic MT bundles apply forces to the nucleus during unperturbed conditions [29] and they can efficiently recenter nuclei in small and large cells after their experimental displacement [31,36]. Importantly, even under this dramatic condition, cytoplasmic MTs push the nucleus producing severe deformations on the NE, but maintaining the nuclear integrity and cell viability intact [36]. This suggests that yeasts as animal cells have mechanisms to maintain nuclear homeostasis under severe mechanical stress conditions.

### Chromatin Tethers to the NE Influence the Mechanical Response of the Nucleus

In the last few years, chromatin has emerged as an important element that contributes to the mechanical resistance of the nucleus [75,76]. Forces applied to isolated fission yeast nuclei, as well as forces produced by MTs on the nucleus of live cells, are transmitted to the NE producing NE deformations. In the range of forces similar to those estimated for MT dynamics (3–4 pN per bundle), chromatin tethers to the NE through Ima1, Man1, and Lem2 restrict chromatin flow and the mechanical response of the NE is elastic, so it is able to respond and recover the initial state quickly once the force ceases [77]. In the absence of chromatin tethers (INM mutants), chromatin flow is increased and NE deformations now show a slower recovery to MT-dependent fluctuations. Interestingly, in *S. pombe* cells, NE fluctuations in response to MT forces are more prominent in the area close to the SPB, which suggests that this area of the NE is subjected to stronger MT forces. Accordingly, impairment of these SPB-enriched chromatin tethers in the double mutant *lem2Δ ima1Δ* results in the most pronounced and lasting NE deformations in vivo. This suggests that the regulation of chromatin tethers at the NE might constitute an active mechanism to modulate chromatin flow and the mechanical response of the nuclei to cytoplasmic MT-driven forces [77]. Of note, underneath the SPB are attached the largest blocks of centromeric heterochromatin of the three chromosomes. As *lem2* deletion also affects centromeric chromatin silencing [53] and therefore the state of chromatin compaction, this likely contributes to the altered mechanical response of the nucleus in this mutant. In addition, Lem2 has been recently shown to function as a barrier for membrane flow between the NE and other parts of the cellular membrane system [78]. The *lem2Δ* mutant shows altered nuclear membrane lipid composition [78,79], that might modify membrane tension [80,81]. This points to Lem2 as an interesting candidate to modulate the mechanical properties of the nucleus at different levels in response to MT forces. In animal cells, physical forces that deform the nucleus can produce transient NE ruptures that are frequently accompanied by DNA damage [82,83,84,85]. Thus, a proper mechanical response of the nucleus to perturbation is critical for genome integrity.

**Figure 3:**
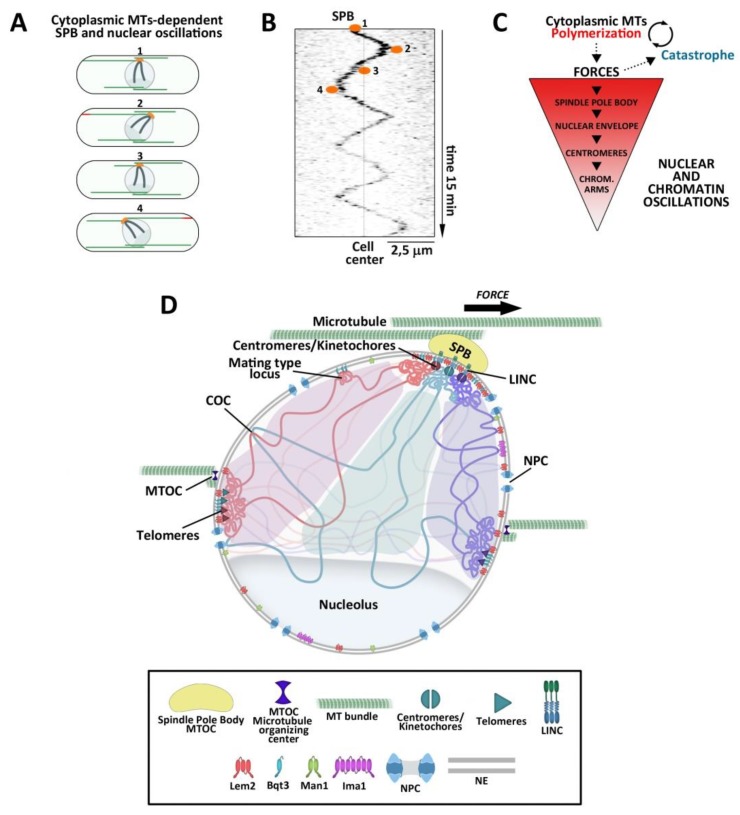
Fission yeast nucleus-SPB and NE under the forces produced by MTs. (**A**) Schematic representation of a fission yeast cell over time. The nucleus suffers periodic oscillations. Chromosomes are depicted as dark lines. SPB/centromeres are depicted in orange. MTs are depicted in green. The red color at MT ends represent stronger forces. (**B**) Image of the SPB (marked with GFP) showing regular oscillations around the cell center. Numbers correspond to those marked in A. Nuclear and SPB oscillations depend on alternative MT pushing (by polymerization) at each cell tip. (**C**) Schematic representation of the transmission of forces produced by cytoplasmic MTs to the chromatin, coupled to MT dynamics. (**D**) Schematic representation of a fission yeast nucleus under MT-dependent forces applied to the SPB, NE, and centromeric regions. Tethering of chromatin to the NE through INM proteins contributes to support nuclear mechanics. Notice that forces produced by other non-SPB MT bundles at other sites of the NE are not shown.

Importantly, interphase MT depolymerization is thought to be regulated by force- and length-dependent mechanisms [36,86,87,88]. When a growing MT bundle contacts the cell tip and keeps polymerizing, it generates forces that build up at the contact site. This promotes MT depolymerization that in turn, releases the tension in the NE [36,87] (schematized in Figure 3). In this way, MT regulation might act as a negative feedback mechanism during nuclear mechanics. Whether the regulation of membrane flow and MT dynamics are part of a cellular response to mechanical stress is an interesting question to be addressed in the future.

## 5. Effect of MT Cytoskeletal Forces on Chromatin Dynamics

In *S. pombe*, the use of in vivo genomic tagging systems such as the *lacO*/LacI-GFP or *tetO*/TetR–tdTomato systems have allowed the study of the behavior of specific chromatin positions in relation to MT movements. These studies have shown that cytoplasmic MTs affect chromosome behavior both during the meiotic and the mitotic cell cycles [32,33,34,35]. 

### 5.1. MTs and Dynein Modulate the Extent of Chromatin Contacts During Meiotic Prophase

In *S. pombe*, the effect of MT-driven movements on chromatin dynamics has been best characterized during meiosis. During meiotic prophase, rapid and extensive nuclear movements are driven by dynein- and MT-dependent pulling forces generated at the cell tips during the so-called “horsetail movement” [89,90,91,92,93]. These forces are transmitted from the MTs in the cytoplasm to the chromatin through the SPB and LINC complexes at the NE as happens during interphase [32,92,93]. However, during horsetail, chromosomes display a bouquet configuration in which telomeres are clustered and attached to the SPBs through LINC complexes, whereas centromeres are distantly positioned relative to the SPB and free in the nucleoplasm [94,95]. Thus, MT-generated forces are transmitted to telomeres and they have also been shown to affect the behavior of distant loci at chromosome arms [32]. It is known that mutations in dynein that abolish nuclear movement during meiotic prophase result in unpaired chromosomes and reduced recombination [91,96]. Indeed, it has recently been shown that during meiosis homologous loci display cycles of pairing and unpairing (“chromosome breathing”) that are the result of chromatin stretching and relaxation respectively, due to dynein and MT-dependent nuclear oscillations. These nuclear oscillations are required for the initial pairing of homologous loci during meiotic prophase [32]. After that, dynamic chromosome stretching and relaxation result in continuous cycles of pairing and unpairing of homologous loci that avoid prolonged association of chromosomes. Chromosome pairing at homologous sequences promotes recombination and inhibition of nuclear oscillations by MT depolymerization in cells where the loci were already paired, results in permanent association of the homologous loci, even after restoring the MT cytoskeleton. This permanent association is the result of the accumulation of irresolvable recombination intermediates and leads to chromosome mis-segregation at meiosis I [32].

Therefore, meiotic MT-driven nuclear movements promote dynamic chromosome pairing and unpairing to modulate the extent of chromatin contacts and recombination. The lack of MT-dependent movements of chromosomes during meiotic prophase affects spore viability and the efficiency of gamete production [32]. Analogously, in animal cells, MTs, MT motors and LINC complexes collaborate to produce nuclear rotations at meiotic onset that are required for chromosome pairing, clustering, and synapsis [97]. Interestingly, during these nuclear movements, the LINC complex and the mitotic kinase NuMA are required to maintain the integrity of the NE in these conditions of mechanical stress [97].

### 5.2. Interphase MT Movements Promote the Repair of Persistent DSBs

The NE constitutes a protective environment where persistent double-strand breaks (DSBs) are recruited and repaired [98]. In *S. cerevisiae* and *Drosophila* cells, this relocalization requires proteins of the SUN family [99,100] and it has been shown to promote alternative HR-mediated repair pathways [100,101,102].

In *S. pombe*, the LINC complex also contributes to the repair of persistent DSBs [34]. The induction of DSBs leads to the formation and local concentration of Sad1 and Kms1-containing foci at the NE to which DSBs are recruited. Upon persistent DNA damage, these foci coalesce at the SPB, bridging in this way the DSB with cytoplasmic MTs and increasing DSBs mobility as they follow MT-driven SPB oscillatory movements (Figure 2). Disruption of DSB connection to the MTs by deleting *kms1*, or *mto1*, a cytoplasmic factor that mediates the nucleation and attachment of cytoplasmic MTs to the nucleus [30,103,104,105,106,107], leads to decreased efficiency of HR-based DNA repair response [34]. This suggests that MT-driven movements promote HR-based DNA repair. Thus, persistent and/or irresolvable DSBs are recruited to the nuclear periphery and to the SPB by LINC complexes to increase their mobility and the chance to find a new donor sequence and/or to promote alternative repair pathways [34]. Budding yeast chromosomes not only increase their mobility at sites of DSBs, but they also increase their global mobility upon DSB induction and this depends on MT dynamics [108,109].

### 5.3. Interphase Chromosomal Movements Affect Distribution of Cohesin Into Chromosomes and the Efficiency of DNA Repair 

In the fission yeast, the magnitude of nuclear movements during the mitotic cycle is much smaller than during the meiotic horsetail period. Nonetheless, during interphase, cytoplasmic MT bundles also move chromosomes in an oscillatory manner via linkages through the NE at the SPB and other distant sites [35] (Figure 3A–C). During S/G2, these movements lead to cycles of association and disassociation of sister loci that have been referred to as “chromatid breathing”. Chemical disruption of MTs or deletion of *mto1* abolishes chromosome movements and results in altered cycles of chromatin breathing in which chromatids appear more frequently unpaired compared to unperturbed cells [35]. This phenotype is indicative of decreased sister chromatid cohesion [110,111,112] as it is phenocopied in *psc3-1T* cohesin mutant [35]. *mto1Δ* mutant cells, in which MT-driven movements of chromatin are abolished, show a significant decrease in the efficiency of intrachromosomal HR. Consistently, *mto1Δ* cells present increased sensitivity to DNA-damaging agents and defects in HR-based DNA repair [35]. Of note, the effect of MT movements on chromatid breathing is specific for loci distant from centromeres, suggesting that MT movements affect cohesin distribution specifically at chromosome arms and not at centromere-proximal loci. Consistently, the levels of Rad21 cohesin bound to centromeres are not affected in *mto1Δ* compared to wild-type cells, whereas the levels of Rad21 bound to several other loci at different positions of chromosome arms are significantly reduced [35]. Mto1 regulates cytoplasmic MTs and is not detected inside the nucleus [35]. This suggests that its function in chromatid cohesion is likely due to its effects on cytoplasmic MT dynamics. Therefore, cytoplasmic MT-driven chromatin movements affect directly or indirectly the distribution of cohesins specifically onto chromosome arms, and this results in defective HR-based DNA repair. 

## 6. Concluding Remarks

During the past years, the fields of nuclear organization and mechanics have witnessed remarkable progress and have contributed to a better understanding of how genomes are organized in the interphase nucleus, how this is critical for genome functions, and how forces applied to the nucleus can alter this organization and regulate cellular functions. Mechanical forces are intrinsic to many cellular processes. For example, fluid forces and shear stress are known regulators of cardiac development [113,114] or immune system function as forces influence leukocyte differentiation, migration, and invasion [115,116]. The cytoplasmic microtubule cytoskeleton is gaining much attention as a force-producing structure that is able to influence nuclear functions during interphase. MTs, MT motors, and LINC complexes are required for nuclear migration that occurs for instance during mammalian brain or skeletal muscle development [117,118]. During brain development, neuronal migration requires the repetitive formation of a long cellular projection and the subsequent MT-dependent migration of the nucleus into this projection. During this migration, MTs produce pulling forces that result in reversible local deformations of the NE at the sites of MT contacts [118] showing that cells have mechanisms to bear physiological levels of mechanical stress on the nucleus. Proper chromatin conformation and tethering to the NE, nuclear membrane dynamics, and regulation of MT dynamics are emerging as important pathways that might collaboratively regulate the forces produced on the NE and the chromosomes. To understand how the nucleus resists and responds to MT-dependent forces, how MTs are regulated accordingly, and how force produced by cytoplasmic MTs on the nucleus might in turn regulate nuclear processes will be exciting areas of future research. The relative complex nuclear architecture of fission yeast cells and its MT organization and connections with the NE and chromatin make this yeast a useful system to address these questions. 

## Abbreviations

NE: Nuclear envelope, INM: Inner nuclear membrane, ONM: Outer nuclear membrane, NPC: Nuclear pore complex, SPB: Spindle pole body, MT: Microtubule, MTOCs: Microtubule organizing centers, LINC: Linker of nucleoskeleton and cytoskeleton, LEM: Lap-emerin-Man1, HR: Homologous recombination, SUN: Sad1/UNC-84, KASH: Klarsicht/ANC-1/Syne homology, MSC: MAN1/Src1, pol III: Polymerase III, LTR: Long terminal repeat, COC: Chromosome organizing clump, TFIIIC: Transcription factor for polymerase IIIC, BEs: Boundary elements, DSBs: Double-strand breaks, ETC: Extra-TFIIIC site.
